# Draft Genome Sequence of *Pseudomonas aeruginosa* ATCC 9027 (DSM 1128), an Important Rhamnolipid Surfactant Producer and Sterility Testing Strain

**DOI:** 10.1128/genomeA.01259-15

**Published:** 2015-10-29

**Authors:** Anne Mai-Prochnow, Mark Bradbury, Anthony B. Murphy

**Affiliations:** a CSIRO Manufacturing, Lindfield, New South Wales, Australia; b CSIRO Food and Nutrition, North Ryde, New South Wales, Australia

## Abstract

*Pseudomonas aeruginosa* ATCC 9027 (DSM1128) is often used as a quality-control strain for sterility and microbial contamination testing and is an important biosurfactant producer. Here, we present the 6.4-Mb draft genome sequence and highlight some genomic differences to its closest relative, *P. aeruginosa* strain PA7.

## GENOME ANNOUNCEMENT

*Pseudomonas aeruginosa* is a versatile Gram-negative rod that is found in soil, water, human flora, and most man-made environments throughout the world. It is capable of causing disease in animals, including humans. *P. aeruginosa* ATCC 9027 was originally isolated from an outer-ear infection by C. P. Hegarty in 1943. It is today frequently used as a quality-control strain in sterility and contamination assays. In addition, *P. aeruginosa* ATCC 9027 is an important producer of the biosurfactant rhamnolipid (1). Rhamnolipid produced by *P. aeruginosa* is the most studied microbial surfactant for bioremediation. Most recently, the strain has been used for testing bacterial biofilm resistance to treatment with cold atmospheric-pressure plasma (2).

While *P. aeruginosa* ATCC 9027 is a widely used, important strain, its complete genome sequence had not been determined. Some single genes of *P. aeruginosa* ATCC 9027 have been sequenced, including some housekeeping genes (3), and it was found that it exhibits high sequence diversity in several analyzed genes (4, 5). *P. aeruginosa* ATCC 9027 appears to be closely related to the taxonomic outliner *P. aeruginosa* PA7 (3).

In our study, genomic DNA of *P. aeruginosa* ATCC 9027 was isolated using the DNeasy Blood and Tissue kit (Qiagen) and purified using the Wizard SV Gel and PCR cleanup system (Promega). DNA sequencing was performed at the Ramaciotti Centre for Genomics (UNSW, Australia) using Illumina MiSeq technology. A 6,362,326-bp assembly with ~180× coverage was constructed using the A5-miseq pipeline version 20140113 (6). The assembly consists of 80 contigs (>500 bp), with a mean GC content of 66.6%. The annotation was performed with the NCBI Prokaryotic Genomes Automatic Annotation Pipeline (http://www.ncbi.nlm.nih.gov/genome/annotation_prok), which predicted a total of 5,772 protein-coding genes and 58 tRNAs. The *N*_50_ and *N*_90_ of the assembly were 294,007 bp and 665,70 bp, respectively.

The closest relative of *P. aeruginosa* ATCC 9027 appears to be *P. aeruginosa* PA7, a nonrespiratory isolate with a range of antibiotic resistances (3). The draft genome of *P. aeruginosa* ATCC 9027 does not have a number of genes related to antibiotic and heavy-metal resistance that are present in *P. aeruginosa* PA7, notably UV-light resistance protein B, putative mercury resistance protein, streptomycin 3′-kinase, bleomycin resistance protein, kanamycin kinase type II, aminoglycoside 3′-phosphotransferase, and streptomycin phosphotransferase. The absence of these genes may account for the unique resistance pattern, which has implications for the use of the strain in sterility and contamination assays.

In addition to differences in resistance genes, *mucA*, a negative regulator of the sigma factor AlgU is absent in *P. aeruginosa* ATCC 9027. MucA plays a role in the differentiation of *P. aeruginosa* into the alginate-producing form. Inactivation of mucA (such as through the absence in *P. aeruginosa* ATCC 9027) has been shown to induce a switch from the nonmucoid to mucoid state, resulting in the constitutive expression of alginate biosynthetic genes (7). The production of rhamnolipid, alginate, and resulting mucoid phenotypes all contribute to biofilm formation and virulence in *P. aeruginosa*.

### Nucleotide sequence accession numbers. 

This whole-genome shotgun project has been deposited at DDBJ/EMBL/GenBank under the accession number LJGL00000000. The version described in this paper is the first version, LJGL01000000.

Unassembled reads are also available from the NCBI short-read archive (SRA) under the accession number SRR2043045.
